# Interpersonal violence and substance use: mental health and self-harm/suicide among Mexicans in VDS-UMS in the United States

**DOI:** 10.3389/fpubh.2026.1975732

**Published:** 2026-10-08

**Authors:** Silvia Morales-Chainé, Alma Luisa López-Fuentes, Gisell Atziri Duarte-Martínez, Isaura Angélica Lira-Chávez, Gonzalo Bacigalupe, Rebeca Robles-García, María Elena Medina Mora, María Gudelia Rangel-Gómez

**Affiliations:** 1 Facultad de Psicología, Universidad Nacional Autónoma de México, Mexico City, Mexico; 2 United States-Mexico Border Health Commission (Mexican Section), Tijuana, Mexico; 3 Department of Counseling, School Psychology, and Sport, College of Education and Human Development, University of Massachusetts Boston, Boston, MA, United States; 4 Centro de Investigación en Salud Mental Global INPRFM-UNAM, Instituto Nacional de Psiquiatría Ramón de la Fuente Muñiz, Mexico City, Mexico

**Keywords:** interpersonal violence victimization, lifetime alcohol and substance use, mental health, Mexican immigrants, self-harm-suicide thoughts and behaviors

## Abstract

**Background:**

Interpersonal violence victimization (IVV) is associated with adverse mental health outcomes, including substance use, self-harm, and suicidal thoughts and behaviors. This study examined the relationships among these conditions in Mexican immigrants living in the United States, stratified by sex and mental health treatment-seeking status.

**Methods:**

We conducted a cross-sectional study of 896 Mexican immigrants residing in the United States. Participants were predominantly women (72%), and 71% were seeking mental health treatment. Structural equation models were estimated for the overall sample and separately by sex and treatment-seeking status.

**Results:**

IVV was associated with posttraumatic stress symptoms and substance use. Substance use and depressive symptoms were, in turn, associated with self-harm and suicidal thoughts and behaviors. These associations were consistent across sex and treatment-seeking groups. Substance use contributed more strongly to the model among men, whereas posttraumatic stress symptoms contributed more strongly among participants seeking mental health treatment.

**Conclusion:**

Mexican immigrants visiting the Ventanillas de Salud and Mobile Health Units (VDS-UMS) reported psychoactive substance use associated with interpersonal violence victimization and self-harm and suicidal thoughts and behaviors, whereas depressive symptoms were independently related to suicide risk. These findings support integrated screening and treatment strategies addressing violence exposure, substance use, and mental health symptoms among Mexican immigrants served through these programs in the United States, all within the context of stressors that interact with vulnerability associated with migration.

**Clinical implications:**

Integrating screening and early intervention for interpersonal violence victimization and substance use within existing binational community mental health programs may reduce self-harm and suicidal behaviors among Mexican immigrants.

## 1 Introduction

Migration often exposes individuals to multiple forms of interpersonal violence that threaten both physical safety and psychological well-being. Among Mexican immigrants living in the United States, interpersonal violence victimization (IVV) has been associated with substance use and a range of adverse mental health outcomes, including depression, anxiety, posttraumatic stress disorder (PTSD), and suicidal thoughts and behaviors (1). Understanding how these conditions are interrelated is critical for developing effective prevention and treatment strategies for the immigrant population.

IVV is associated with depression, anxiety, posttraumatic stress symptomatology, and harmful alcohol and other substance use (SU) in Latino immigrants (2). Some years ago, Donlan and Lee (3) also reported high indicators of suicidal ideation and thoughts of self-harm and emotional symptoms such as unhappiness among Mexican immigrants in the United States Kim et al. (4) recently described self-harm and suicidal thoughts and behaviors (STBs) in Latino immigrants in the United States due to IVV. They also reported that men were more likely to use substances, whereas women were more likely to report mental health symptoms.

Among migrant and refugee women, Taiebine (5) already explained that gender-based violence is related to the stress of displacement, insecurity, and inadequate healthcare access, which may exacerbate existing mental health problems or contribute to the onset of new ones. Among migrant Latino populations, Rai et al. (2) also suggested that interpersonal violence may constitute a stressor that, in interaction with conditions of vulnerability associated with migration, increases the risk of mental health deterioration. Sustained exposure to violence and social and structural stressors may generate complex psychological needs and create conditions in which substance use is employed as a coping strategy, thereby increasing both mental health and substance use-related risks.

Because of the relationship between IVV, SU, mental health symptoms, and STBs among Mexican immigrants in the United States, we have provided primary mental health care to our community through the Health Windows and Mobile Health Units Program (Ventanillas de Salud y Unidades Móviles; Spanish acronym, VDS-UMS; (6)). The program has allied with several United States-based agencies to reduce the mental health treatment gap for Mexicans in the United States. The program provides routine screening for substance use, exposure to violence, and common mental health conditions, followed by brief psychological interventions or referral for specialized care when indicated.

Immigrants are at higher risk of harm to their physical and emotional integrity (7). Violence may become normalized throughout the migration process, increasing cumulative exposure to traumatic experiences. Phillimore et al. (8) have described normative routines and acts of violence that constitute multiple forms of IVV while a person migrates between countries. For instance, everyday experiences of denial of healthcare are slow forms of violence and abandonment described as part of experiencing IVV, in the same way as physical, emotional, and sexual violence.

Based on a systematic review, Gonzalez et al. (9) reported IVV among Latinas in the United States, with 41 studies reporting the highest prevalence for Mexicans. Mexican immigrants are used to dealing with violence, abuse, discrimination, and stigma (10). In a non-immigrant clinical sample, Hindmarch et al. (11) described how IVV is associated with SU and mental health symptoms. The authors reported that 40% of their sample, mainly female and youth participants from a 797-patient sample in primary care, experienced IVV. Hindmarch et al. (11) defined IVV as sexual, physical, and domestic violence, with fewer women experiencing physical violence and more participants with SU reporting violent events such as physical or sexual assaults. According to Reyes et al. (12), this type of violence is also associated with mental health symptoms during mobility in Mexican immigrants. Borges et al. (13) found that United States-born Mexican Americans and Mexican-born immigrants were both at higher risk for sexual victimization, while those with mental disorders were more likely to suffer any form of violence, regardless of whether they had undergone the immigrant experience.

Given that violence among individuals, who may or may not know each other, includes a range of acts, IVV comprises physical, emotional, and violent sexual acts experienced inside or outside the family (14, 15). Sabina et al. (16) have evaluated IVV, also defined as physical assaults, threats, and sexual assault among Latino women seeking help and living in high-density neighborhoods in the United States Taiebine (5) described how gender-based violence against immigrant women can have significant consequences for both brain and mental health, including the neurological effects of trauma and the development of conditions such as posttraumatic stress disorder, anxiety, and depression. The effects may also impair cognitive functioning and emotional regulation and negatively affect interpersonal relationships. Notably, interpersonal violence is embedded in policies providing legal status and protection for unjust practices while depriving certain groups of individuals of their inalienable human rights in a context of barriers to healthcare services (17, 18).

According to Gonzalez et al. (9), Mexicans are the largest group of Latino immigrants reporting IVV (90%) in the United States Sabina et al. (16) reported that those seeking help after IVV and depressive symptomatology were mostly Latino women. In our attempt to better understand IVV, we examined studies reporting intimate violence victimization, most of which also involved Latino women. However, González et al. (2020) concluded that the prevalence of IVV among Latino men and women in the United States remained unclear and should be continuously monitored.

A broad range of prevalence estimates is available for lifetime and past-year intimate violence victimization among the undocumented population in the United States (13.9 to 93% and 3.8 to 46.9%, respectively; (19)). Study samples were drawn from citizens of Asian and other countries in the United States, including two studies of Mexican immigrants. Intimate violence victimization (whether physical, psychological, or sexual) has been found to be associated with mental health outcomes (12). Depression appears to be the most prevalent mental health outcome, followed by anxiety, posttraumatic stress disorder (PTSD), and SU among Latino women exposed to intimate violence in the United States. In this context, Reyes et al. (12) have recommended researching not only the nature of the association between IVV and mental health conditions, but also suicidal ideation and substance use disorders among Latino men, not just women, in the United States.

The current cross-sectional study explores the relationship between IVV, SU, mental health symptoms, and STBs in a group of Mexican immigrants seeking help at the health windows of Mexican Consulates in the United States due to mental disorders between February 15 and December 11, 2025. Mexican immigrants exposed to IVV may enter the study with accumulated psychological distress that has not previously received formal attention, while coping may occur through family networks, informal resources, avoidance, or, in some cases, substance use and STBs. Thus, the hypothesis states that IVV is associated with SU (Ha1) and STBs (Ha2), and the presence of depression, anxiety, and PTSD symptoms (Ha3, Ha4, and Ha5, respectively) among Mexican immigrants in the United States. Efforts by community partners to screen early for IVV, mental health symptoms, and SU and by clinicians to deliver interdisciplinary mental health treatment could help prevent STBs among Mexicans residing in the United States (2, 4, 12). Although most study data are drawn from population or community surveys, this study was conducted among Mexican immigrants seeking help (70.8% of the sample requested treatment and 29.2% requested psychoeducational care). Those seeking help usually report more severe or current disorders than the general population, making it possible to identify problems and treatment needs in a different population at times of high risk for immigrants.

## 2 Methods

### 2.1 Participants and procedure

This study is a cross-sectional analysis of data on 896 Mexicans served by the VDS-UMS alliance in the United States. Of the total sample, 28% were men and 72% were women, aged 18 to 84 years (Mo = 48 years). With regard to marital status, 32.6% were single, 41.9% married, 7.9% in common-law unions, 8.1% divorced, 3.2% widowed, and 6.3% separated. As for employment status, 37.1% of participants were unemployed, and 62.9% were employed. At the time of screening, 18.5% were living in Massachusetts, 16.4% in California, 8.9% in Arizona, 3.9% in Texas, and 52.3% in 20 other states such as New Jersey. A total of 70.8% of participants were seeking mental health treatment, while 29.2% were only seeking psychoeducational information. Participant distribution was not homogeneous in terms of size or sociodemographic background because of the voluntary enrollment. We must therefore consider potential bias due to substantial self-selection among participating Mexican immigrants rather than random community sampling. Accordingly, our sample was an immigrant sample seeking some form of informal or formal psychological care. Distribution of participants by sex and mental health treatment-seeking status is given in Table 1.

**Table 1:** Proportions by sex and mental health treatment-seeking.

Mental health treatment-seeking						
Sex	No		Yes		Total	
	*n*	*%*	*n*	*%*	*n*	%
Men	100	39.8	151	60.2	251	28
Women	162	25.1	483	74.9	645	72
Total	262	29.2	634	70.8	896	100

Participants voluntarily enrolled in a web-based application called Salud Mental Acción Migrante (Mental Health Immigrant Action; https://saludmental.accionmigrante.unam.mx) through a face-to-face invitation by community partners of the VDS-UMS alliance located in Mexican Consulates in the United States (6). The Mexicans contacted agreed to participate in the study between February 15 and December 11, 2025. Once they had submitted their application, they used their email addresses to register and complete an evaluation of their mental health status and receive free feedback and treatment if required.

We used the criteria for internet surveys, such as data protection, development, testing, contact mode, publicizing the survey, compulsory/voluntary participation, completion rate, cookies used, IP check, log file analysis, registration, and atypical timestamp considerations (20). Since the survey platform automatically eliminates respondents who fail to complete the survey, we were only able to obtain complete responses, with no incomplete survey responses. We are therefore unable to describe Mexican immigrants who were not interested in participating in this strategy.

The Ethics Committee of the Psychology Faculty of the National Autonomous University of Mexico (UNAM) approved the study (code FPSI/422/CEI/427/2025). All participants signed the informed consent form explaining that findings would be used for epidemiological research and stating their right to refuse to comply with data requests and withdraw from the study at any point. Although the study did not offer any incentives, it provided written psychological education and feedback with infographics on problem-solving techniques. The feedback contained information on how to obtain remote mental health treatment from public health services if required. Finally, we asymmetrically transformed personal information from the database using alphanumeric codes to protect it and stored it in the official university domain. Only authorized researchers were able to access the information using data protection security locks.

### 2.2 Instruments

The survey includes dichotomous response questions (yes or no) regarding participants’ sex and remote mental health treatment-seeking, as well as five psychological screening self-reports. Self-reports do not constitute formal clinical diagnoses; they are measures concerning common presentations of stress, depression, anxiety, lifetime psychoactive substance use, and suicidal thoughts and behaviors, as recommended by mhGAP (2016) (37). The Life Events Checklist 5th edition (LEC-5; (21, 22)) screens for IVV in the past 6 months. The IVV subscale comprised three dichotomous-response items (yes or no) from the seven-item general scale (see Appendix A). This subscale obtained an *X*^2^ (13) = 103.717, *p* < 0.001, RMSEA = 0.053 (0.44–0.063), CFI = 0.961, and TLI = 0.937, and a Cronbach’s alpha of 0.62 (23). Participants were considered at risk if they reported at least one symptom on the scale.

Based on the IVV event that had bothered each participant most at the time, the Brief Posttraumatic Checklist (PCL-5; (24, 25)) was administered to evaluate the core symptoms of posttraumatic stress disorder (PTSD) in the past month. The checklist contains two B-items for re-experiencing, two C-items for avoidance, two D-items for negative alterations in cognition and mood (NACM), and two E-items for hyperarousal (see Appendix A). The PCL-5 scale obtained an *X*^2^ (152) = 2381.298, *p* < 0.001, RMSEA = 0.077 (0.074–0.080), SRMR = 0.019, CFI = 0.971, TLI = 0.94, and a Cronbach’s alpha of 0.99 (23). Participants screened positive for PTSD when they obtained a score of 14 or more points in addition to having experienced bothersome symptoms for over a month.

STBs were screened through a six-item scale with a dichotomous response format (adapted from the Mental Health Gap Action Program [mhGAP] algorithm, 2016). Items refer to current suicidal thoughts and behaviors (STB) and self-harm thoughts and behaviors (ShTB) (see Appendix A). A factor model obtained an *X*^2^ (33) = 447.803, *p* < 0.001, RMSEA = 0.071 (0.65–0.077), CFI = 0.991, TLI = 0.988, and a Cronbach’s alpha of 0.90 (23). Participants were considered at risk when they had at least one symptom in each scale.

The depression and anxiety screening scales contained three and five items, respectively, based on 11 response options ranging from total absence of the symptom (zero) to total presence of the symptom (10) in the past two weeks (adapted from (26); see Appendix A). Based on a scale with a total score of eight, we obtained an *X*^2^ (406) = 43.509, *p* < 0.001, RMSEA = 0.062, SRMR = 0.046, CFI = 0.933, and TLI = 0.923 (27). Both scales achieved Cronbach’s alpha values of 0.83 and 0.94, respectively. We screened for depression and anxiety by summing the scores and dividing the totals by three and five, respectively, and converting them into percentages. An average score of over 60% on each scale was considered the threshold for each mental health problem.

The WHO Alcohol, Smoking, and Substance Involvement Screening Test (ASSIST; (28)) identifies harmful use of 10 substances (such as alcoholic beverages including beer, wine, and spirits). We used the first question out of eight to screen for lifetime SU, based on a dichotomous yes-or-no response (see Appendix A). The first ASSIST question obtained an *X*^2^ (27) = 100.478, *p* < 0.001, RMSEA = 0.033 (0.026–0.040), CFI = 0.972, TLI = 0.963, and a Cronbach’s alpha of 0.61 (23). We scored SU by adding affirmative answers from nine groups of substances: tobacco, alcohol, cannabis, cocaine, stimulants, inhalants, sedatives, hallucinogens, and other substances. Participants were considered at risk when they had used at least one substance in any group. For all scales, participants’ indices from the confirmatory factor analyses and Cronbach’s alphas are shown in Appendix B.

#### 2.2.1 Data analysis

We conducted confirmatory factor analysis (CFA) for each scale (using the maximum likelihood [ML] and the diagonally weighted least squares [DWLS] procedures (29, 30)). We evaluated the overall fit of the models with the chi-square goodness-of-fit test using the Comparative Fit Index (CFI), the Tucker-Lewis Index (TLI), the Root Mean Square Error of Approximation (RMSEA), and the Standardized Root Mean Square Residual (SRMR). We did not consider the SRMR index for categorical data (29). We calculated the Cronbach’s alpha coefficient for each scale.

We obtained scores for each scale, classifying participants according to whether they met the risk criteria for IVV (LEC-5 subscale), STBs (STB and ShTB mhGAP-based subscales), PTSD (PCL-5), depression and anxiety subscales (adapted from Goldberg’s scales), and SU (from ASSIST). We compared the distribution of participants across these criteria by sex and mental health treatment-seeking status, performing Chi-squared tests and considering *p*-values below 0.05 statistically significant.

We used structural equation modeling (SEM) to evaluate theoretically specified associations among IVV, SU, mental health symptoms, STB, and ShTB using a combination of continuous and categorical observed indicators (29, 30). The IVV indicators, STB and ShTB indicators, and lifetime SU indicators were specified as ordered categorical variables. Because these indicators were dichotomous, their thresholds were estimated, and associations between pairs of categorical indicators were represented by tetrachoric correlations. Depression, anxiety, and PTSD indicators were treated as continuous. Accordingly, associations between continuous indicators were based on Pearson correlations, whereas associations between continuous and ordered categorical indicators were represented by polyserial correlations. Given the inclusion of categorical indicators, the SEM was estimated using DWLS with the delta parameterization and robust standard errors.

The hypothesized ordering of variables was specified *a priori* on the basis of theoretical considerations and previous empirical evidence regarding the relationships among exposure to IVV, mental health symptoms, SU, and STB/ShTB. Because the data were cross-sectional, the directional paths specified in the models were not interpreted as evidence of temporal or causal directionality. Rather, SEM was used to determine whether the hypothesized structural pathways were consistent with the observed pattern of associations. We evaluated the overall model fit using the chi-square test and complementary goodness-of-fit indices, including the CFI, TLI, and RMSEA. We compared nested models to determine whether the more parsimonious specification provided a fit comparable to that of the more complex model (30). The complex model estimated 12 unknown parameters, whereas the parsimonious model estimated 10 unknown parameters, based on 10 observed variables and their 28 correlations as known parameters (31). We considered changes in CFI of 0.010 or less and RMSEA of 0.015 or less as evidence that the more constrained model did not substantially worsen model fit (32). We additionally evaluated the overall model by sex and mental health treatment-seeking status, and the model fit across these analyses using CFI and RMSEA. We also assessed the standardized residuals from the covariance matrix to identify localized areas of model misfit (30). Accordingly, we interpreted the resulting structural pathways as theoretically informed associations compatible with the observed cross-sectional data rather than as evidence of causal mediation. For the analysis, we used the Lavaan package (version 0.6-11) from the RStudio® statistical software 2022.02.0 (33) and SPSS Statistics 25 (34).

## 3 Results

### 3.1 Confirmatory factor analyses and Cronbach’s alpha

Appendix B shows the psychometric properties of the scales in the study, as a replication of previous studies (such as (23, 27)). It presents the CFAs of the LEC-5 IVV subscale, the mhGAP STB and ShTB, Depression and Anxiety subscales, and the PCL-5 PTSD Brief Version and the ASSIST SU scale. Model fit indices showed CFIs and TLIs >0.975, RMSEAs <0.059, and SRMRs <0.015 (for continuous data) for all scales. Scale reliability ranged from 0.53 for IVV to 0.97 for the PTSD scale. In this context, the three-item IVV measure showed relatively low conventional internal consistency (reliability = 0.53). This coefficient should therefore be interpreted cautiously, particularly because the IVV indicators were dichotomous and differed in their endorsement thresholds. However, in the SEM, IVV was not represented as an error-free observed composite; rather, it was specified as a latent construct measured by three ordered categorical indicators and estimated using DWLS. The standardized factor loadings were 0.567, 0.681, and 0.705, indicating that two indicators showed relatively strong relationships with the latent IVV construct, whereas the first indicator showed a more modest loading.

### 3.2 Overall risks by sex and mental health treatment-seeking status

Distribution of participants at risk for IVV, STB, ShTB, mental health problems (depression, anxiety, and PTSD), and SU in the total sample, by sex, and by mental health treatment-seeking status is given in Table 2. A total of 34.4% of Mexican participants reported IVV. Six percent of participants were at risk for STB, and 8.6% for ShTB. A total of 18.0% of Mexican participants were at risk for depression, 33.9% for anxiety, and 41.1% were experiencing PTSD. Lastly, 70.2% reported SU.

**Table 2:** Distribution of participants at risk for interpersonal violence victimization, self-harm and suicidal thoughts and behaviors, depression, anxiety, PTSD symptoms, and SU for the total sample, by sex, and by mental health treatment-seeking group (seeking or not seeking).

Interpersonal violence victimization									
Total		Men		Women		No		Yes**	
*n*	%	*n*	%	*n*	%	*n*	%	*n*	%
308	34.4	81	32.3	227	35.2	63	24	245	38.6

STB									
Total		Men**		Women		No		Yes	
*n*	%	*n*	%	*n*	%	*n*	%	*n*	%
54	6	25	10	29	4.5	12	4.6	42	6.6

ShTB									
Total		Men*		Women		No		Yes	
*n*	%	*n*	%	*n*	%	*n*	%	*n*	%
77	8.6	29	11.6	48	7.4	17	6.5	60	9.5

Depression									
Total		Men		Women		No		Yes**	
*n*	%	*n*	%	*n*	%	*n*	%	*n*	%
161	18	49	19.5	112	17.4	32	12.2	129	20.3

Anxiety									
Total		Men		Women		No		Yes**	
*n*	%	*n*	%	*n*	%	*n*	%	*n*	%
304	33.9	80	31.9	224	34.7	63	24	241	38

PCL-5									
Total		Men		Women**		No		Yes**	
*n*	%	*n*	%	*n*	%	*n*	%	*n*	%
368	41.1	86	34.6	282	43.7	76	29	292	46.1

SU									
Total		Men**		Women		No		Yes	
*n*	%	*n*	%	*n*	%	*n*	%	*n*	%
629	70.2	209	83.3	420	65.1	192	73.3	437	68.9

Total sample *n* = 896 Mexican immigrants. Adapted with permission from Morales-Chainé et al. (23). *n*, number of participants; %, percentage of participants; STB, suicidal thoughts and behaviors; ShTB, self-harm thoughts and behaviors; PCL-5, posttraumatic checklist 5th version; PTSD, posttraumatic stress disorder; SU, lifetime substance use; *p < 0.05 and **p < 0.001 indicate significant differences between groups. At-risk group criteria were included in the Instruments section. These criteria were as follows: (1) a score over one for interpersonal violence, STB, ShTB, and SU; (2) an average of over 60% for depression and anxiety; and (3) a score over 14 for the Posttraumatic Checklist.

Table 2 also indicates similar levels of IVV for men and women, yet higher levels in those seeking mental health treatment (38.6%). More men reported ShTB (11.6%) and STB (10.0%) than women (7.4 and 4.5%, respectively; *p* < 0.001); however, no differences in ShTB or STB observed by mental health treatment-seeking status. There were similar proportions of men and women with depression and anxiety symptoms, but more participants seeking mental health treatment had depression (20.3%) or anxiety (38.0%; *p* < 0.001) than participants who were not seeking help. More women reported PTSD symptoms (43.7%) than men (34.6%; *p* < 0.001), and more participants seeking mental health treatment (46.1%) than those who were not (29.0%; *p* < 0.001) reported PTSD common presentations. Finally, more men reported SU (83.3%) than women (65.1%; *p* < 0.001), with no differences being observed in SU by mental health treatment-seeking status.

### 3.3 Structural equation modeling

The parsimonious model was estimated using 10 observed variables and 28 correlations, with a combination of continuous and categorical variables from the total sample. The model yielded *χ*^2^ (533) = 992.117, *p* < 0.001, CFI = 0.933, TLI = 0.925, and RMSEA = 0.031, 95% CI [0.028, 0.034]. The alternative model yielded *χ*^2^ (538) = 1066.439, *p* < 0.001, CFI = 0.923, TLI = 0.915, and RMSEA = 0.033, 95% CI [0.030, 0.036]. We retained the parsimonious model because differences in CFI and RMSEA between the models were within the prespecified thresholds (ΔCFI ≤0.010 and ΔRMSEA ≤0.015; (32)). Thus, the more parsimonious specification represented the observed associations without a meaningful deterioration in model fit. Figure 1 presents the standardized pathways for the overall model. Appendix A presents the factor loadings for the observed indicators, all of which were greater than 0.567, and Appendix C presents the standardized residuals used to identify localized areas of model misfit.

**Figure 1:**
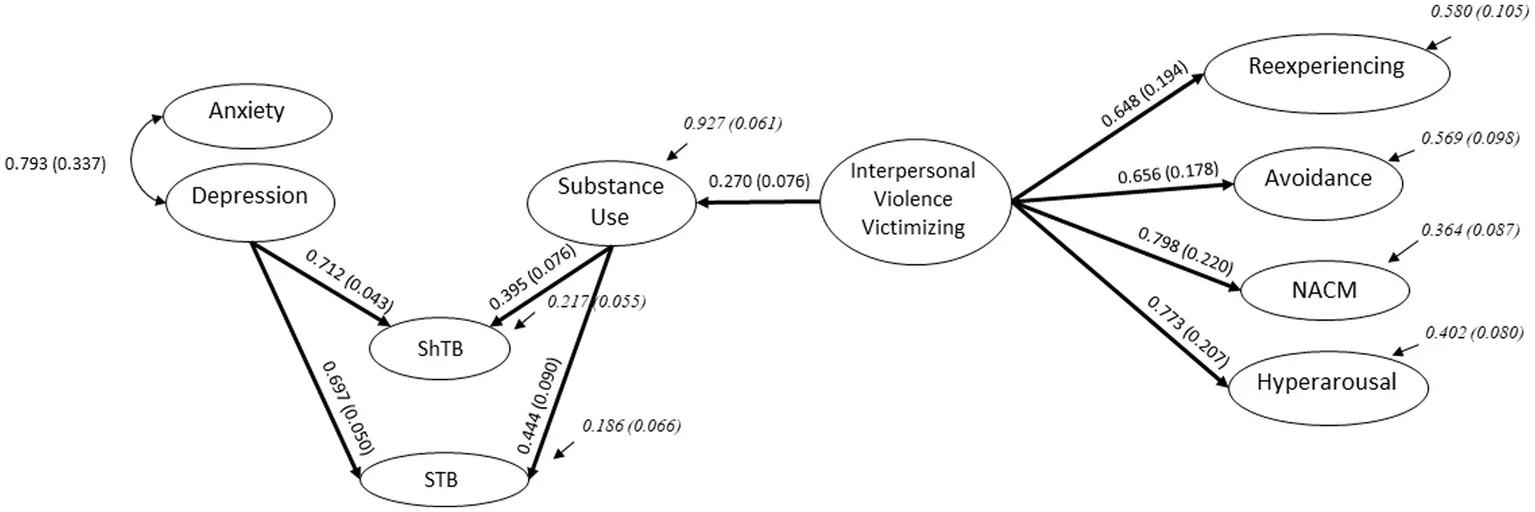
Variables from structural equation modeling (SEM), path coefficients, and residual variances for the whole sample. Adapted with permission from Morales-Chainé et al. (23). STB, suicidal thoughts and behaviors; ShTB, self-harm thoughts and behaviors; NACM, negative alterations in cognitions and mood.

Figure 1 shows that IVV was positively associated with all four PTSD common presentations, with standardized coefficients ranging from *β* = 0.548 to *β* = 0.798. The strongest associations were observed for negative alterations in cognition and mood (NACM; *β* = 0.798) and hyperarousal (*β* = 0.773), followed by avoidance (*β* = 0.656) and re-experiencing (*β* = 0.548). This pattern indicates that greater exposure to IVV was strongly associated with the cognitive, emotional, and arousal-related dimensions of posttraumatic stress. IVV was also positively associated with SU (*β* = 0.270), although this association was smaller than those observed between IVV and PTSD symptoms.

SU, in turn, was positively associated with both STB (*β* = 0.444) and ShTB (*β* = 0.395). Thus, SU was associated with greater levels of suicidal and self-harm thoughts and behaviors. Depression showed even stronger associations with these outcomes: *β* = 0.697 for STB and *β* = 0.712 for ShTB. Taken together, the pattern of standardized coefficients suggests that depressive common presentations were particularly salient in relation to STB and ShTB, whereas IVV was most strongly associated with posttraumatic stress symptoms. Although the factor loadings were clearly established for each group of common presentations, we should carefully consider the symptom “I want to hurt myself” from the depression scale, which may overlap with the ShTB items and explain the high correlation between the two factors. Finally, SU occupied an intermediate position in the hypothesized structural model, being associated with IVV and, separately, with STB and ShTB. However, because the data were cross-sectional, these pathways represent theoretically specified associations and should not be interpreted as evidence of temporal or causal mediation. Moreover, the strong correlation between depression and anxiety (*r* = 0.793) indicates substantial co-occurrence of these symptom domains within the sample.

Table 3 presents the fit indices and structural patterns for models estimated by sex and mental health treatment-seeking status. Across subgroup analyses, the general pattern observed in the overall sample was maintained: IVV was positively associated with PTSD symptoms and SU; SU was associated with STB and ShTB; and depression was associated with both STB and ShTB. Depression and anxiety were also strongly correlated across the models. These recurring associations suggest that the principal pattern of relationships identified in the overall sample was also observable across sex and treatment-seeking subgroups.

**Table 3:** Variables from structural equation models (SEMs) with path coefficients (*β*) and standard errors (*e*) by sex and mental health treatment-seeking.

Predicted variables	Sex				Mental health treatment-seeking			
	Men		Women		No		Yes	
	*n* = 251		*n* = 645		*n* = 262		*n* = 634	
	*β*	*e*	*β*	*e*	*β*	*e*	*β*	*e*
Interpersonal violence victimization								
Re-experiencing	0.648	0.502	0.652	0.182	0.661	0.354	0.636	0.226
Avoidance	0.648	0.444	0.651	0.172	0.617	0.332	0.677	0.211
NACM	0.790	0.551	0.778	0.206	0.770	0.405	0.814	0.256
Hyperarousal	0.784	0.513	0.767	0.200	0.742	0.375	0.794	0.242
Lifetime substance use	0.422	0.202	0.315	0.081	0.443	0.139	0.204	0.090
Depression								
ShTB	0.596	0.062	0.663	0.059	0.683	0.104	0.715	0.047
STB	0.626	0.064	0.768	0.068	0.757	0.120	0.699	0.053
SU								
ShTB	0.308	0.140	0.400	0.085	0.421	0.173	0.365	0.075
STB	0.341	0.138	0.478	0.113	0.381	0.193	0.419	0.076
Depression ≈ Anxiety	0.746	0.776	0.823	0.360	0.814	0.573	0.788	0.411
Model								
*X* ^2^	688.041		828.521		649.268		815.173	
d*f*	533		533		533		533	
*p*≤	0.001		0.001		0.001		0.001	
RMSEA	0.034		0.029		0.029		0.029	
Confidence interval	0.026–0.041		0.025–0.033		0.020–0.036		0.025–0.033	
CFI	0.971		0.931		0.965		0.938	
TLI	0.967		0.923		0.961		0.931	

*n*, number of participants; *β*, beta; *e*, standard error; NACM, negative alterations in cognition and mood; STB, suicidal thoughts and behaviors; ShTB, self-harm thoughts and behaviors; SU, lifetime substance use; ≈, correlation; RMSEA, root mean square error of approximation; CFI, Comparative Fit Index; TLI, Tucker-Lewis Index.

Nevertheless, the magnitude of the standardized coefficients suggests that some correlates may be more salient within particular subgroups. Among men, the associations involving SU were comparatively prominent, suggesting that substance use may be particularly relevant when characterizing the co-occurrence of violence exposure and STB/ShTB in this subgroup. Among participants seeking mental health treatment, associations involving PTSD common presentations were comparatively stronger, indicating a greater concentration of posttraumatic stress symptomatology in relation to IVV. These subgroup results should be interpreted as descriptive differences in the magnitude of associations rather than evidence that sex or treatment-seeking status causally modifies the pathways.

The parsimonious models for women and participants seeking mental health treatment showed only minimal changes in fit relative to the overall model (ΔCFI ≤ 0.010 and ΔRMSEA ≤ 0.015), indicating that the parsimonious specification retained a comparable level of fit in these subgroups (32). In contrast, the models for men and participants not seeking mental health treatment showed changes exceeding these thresholds (ΔCFI >0.010 and ΔRMSEA >0.015). This pattern suggests that the overall model may represent the observed associations less adequately in these two subgroups and that the relationships among IVV, SU, mental health symptoms, STB, and ShTB may differ in magnitude or configuration. These findings warrant further examination in adequately powered subgroup and longitudinal analyses.

In sum, IVV was most strongly related to PTSD symptomatology; depression was most strongly related to STB/ShTB; SU was related both to IVV and to STB/ShTB; and the relative salience of these variables may differ by sex and treatment-seeking status.

## 4 Discussion

Previous studies have reported that IVV is associated with substance use and mental health symptoms among the Latino community in the United States (9, 13). Our findings suggest that Mexican immigrants seeking health care in the VDS-UMS have experienced IVV related to SU and PTSD symptomatology. They also suggest that SU is particularly related to STBs. Depression was also associated with STBs. Association patterns were replicated by sex and mental health treatment-seeking status. SU made a high contribution to the Mexican men’s associative model, while PTSD symptoms made a major contribution to the Mexican mental health treatment-seeking associative model. These findings point to the need for interdisciplinary psychological interventions to close the mental health treatment gap with the aid of Mexican alliances in the United States.

The present study reports the prevalence of IVV in the sample seeking psychoeducational services. The prevalence estimates are within the range reported by Morrison et al. (19) for the undocumented population in the United States, with a third of our sample reporting IVV according to the screening results. We also described the prevalence of mental health symptomatology and SU in our Mexican immigrant clinical sample, expanding the findings of Rai et al. (2) and Reyes et al. (12) regarding Latino populations in the United States, and Taiebine (5) concerning mental health repercussions among immigrant populations after experiencing IVV. In particular, we found that nearly one in five Mexicans was at risk for depression, one in three for anxiety, four in ten for PTSD symptomatology, and seven in ten for SU. In keeping with Donlan and Lee (3), we found that 6.0% of our sample were at risk for STB and 8.6% for ShTB.

Our results suggest that immigrants seeking help are one of the main Latino populations suffering from IVV in the United States (9). Mexican immigrants reported violence and abuse, consistent with Cuesta-García and Crespo (10), and sexual and physical abuse, as reported by Hindmarch et al. (11). We have mentioned physical, emotional, and sexual acts in keeping with the World Health Organization (15) document and Alexander and Johnson’s (14) definition of IVV. Like Sabina et al. (16), we also observed IVV among Latino women seeking help and living in high-density neighborhoods in the United States. In addition, our findings have expanded the description of the asymmetries between men and women experiencing physical violence, as Hindmarch et al. (11) suggested.

Our findings extend previous reports because we observed the prevalence of several conditions for men and for women within the Mexican immigrant sample seeking our psychoeducational services. In particular, we have described the asymmetries in risk by sex and mental health treatment-seeking status. Women appeared vulnerable to PTSD symptoms, whereas more men reported being at risk of STBs and SU. In this context, we have reported the prevalence of IVV for both men and women as suggested by Gonzalez et al. (9). In addition, Mexican immigrants seeking mental health treatment were found to be at risk for IVV and mental health symptomatology: depression, anxiety, and PTSD. We also expanded findings for IVV, reporting results not only for Latino women but also for Latino men in the United States, particularly Mexicans, and based on their mental health treatment-seeking status.

We have suggested the association between IVV and STBs in our Mexican sample, as Kim et al. (4) proposed for Latino immigrants in the United States generally. Our explorations show that SU was related to IVV and STBs within the context of experiencing PTSD symptoms among Mexican immigrants in the United States. Nevertheless, the observed associations suggest that depression is strongly linked to STBs. Our data support Hindmarch et al.’s (11) considerations regarding the association between SU and violent events such as physical or sexual aggression. We also addressed Reyes et al.’s (12) concerns about exploring the association between IVV and mental health symptoms among Mexicans during mobility in the United States. Lastly, we addressed Kim et al.’s (4) recommendation to explore substance use and suicidal acts, as well as mental health issues in both men and women.

We identified replicated association patterns by sex and mental health treatment-seeking status. Nevertheless, lifetime substance use appeared to contribute to Mexican men’s associative model, whereas PTSD symptoms made a major contribution to the Mexican mental health treatment-seeking pattern of association. This could be due to the experience of different types of violence among both females and males (35). As Reyes et al. (12) suggested, we have expanded knowledge of the association between violence, mental health, substance use, and STBs among the Latino population, particularly in Mexican men and women, and by mental health treatment-seeking status in the United States.

We screened for IVV, SU, mental health symptomatology, and STBs in primary care services provided for Mexican immigrants by VDS-UMS in Mexican consulates in the United States. The results showed that IVV was related to SU and PTSD, common presentations (Ha1, Ha5). We also found an association between depression and STBs. STBs, depression, and anxiety symptoms were reported but not directly associated with IVV in our Mexican sample (Ha2, Ha3, and Ha4). We suggest providing empirical evidence to underline the importance of screening and delivering interdisciplinary primary care to Mexicans coping with psychological distress related to immigrant conditions at Mexican consulates in the United States.

Like other Latin American immigrants, the Mexican immigrants in our sample have been exposed to physical, emotional, and sexual threats. It is essential to address these conditions and their consequences to protect their integrity while they are living in the United States (7). In particular, Taiebine (5, 36) has stated that clinical and neuropsychological care is needed; this recommendation refers to screening, assessment, and intervention supported by access to culturally responsive mental health services that integrate psychosocial support and trauma-informed care for immigrant populations. In this context, our findings provide empirical support for policies that expand access to evidence-based mental health services for Mexican immigrants receiving care through the Mexican consular network in the United States.

This strategy would reduce the routine violence affecting Mexican immigrants. Phillimore et al. (8) recommended promoting healthcare services as a means of counteracting what they called slow violence and abandonment. This includes the violence and abandonment resulting from the unavailability of health services for the Latino population, which disrupt their life opportunities and increase what we have called IVV. It is essential to counter policies that protect unfair practices while depriving people of their inalienable human right to health during mobility (5, 17, 18, 36).

The present findings have implications for clinical practice that extend beyond recommending routine screening for IVV, SU, common mental health presentations, and STBs. For Mexican immigrants, particularly those who are undocumented or otherwise socially marginalized, identification of these conditions should be integrated into culturally and structurally responsive pathways to care. Immigrants exposed to violence may face overlapping barriers to disclosure and treatment, including limited language proficiency, mental health stigma, limited familiarity with available legal and health resources, distrust of institutions, and concerns that seeking formal assistance could have immigration-related consequences. Fear of deportation may be particularly consequential because it can discourage immigrants in irregular situations from reporting violence or approaching formal institutions for assistance (5, 36).

Accordingly, screening for IVV should occur in settings perceived as safe and accessible and should be accompanied by clear information regarding confidentiality, culturally and linguistically appropriate assessment, trauma-informed care, and referral pathways that integrate mental health, substance use, social, community, and legal resources. Whenever possible, services should be available in the preferred language and delivered by culturally responsive professionals or trained community-based personnel, in the way VDS-UMS routinely work. Such an approach is particularly relevant to the pattern observed in the present study, in which IVV was strongly associated with PTSD common presentations and was also associated with SU, while SU and depression symptoms were associated with STBs. Thus, identifying violence without simultaneously addressing the structural conditions that may prevent survivors from safely accessing and remaining in care may limit the clinical value of screening. An integrated, survivor-centered approach may therefore be particularly important for reducing the accumulation of mental health, substance use, and suicide-related risks among Mexican immigrants experiencing social or immigration-related vulnerability.

### 4.1 Conclusion

Nearly one in five Mexican immigrants in our VDS-UMS program was at risk for depression, one in three for anxiety, and four in ten for PTSD symptomatology. Seven out of ten Mexicans reported SU. One in three Mexican immigrants reported IVV; 6.0% of the overall sample were at risk for STB and 8.6% for ShTB. More women were prone to experiencing PTSD symptoms, whereas more men reported being at risk for STB and SU. Mexicans seeking mental health treatment were at risk for IVV and mental health symptomatology: depression, anxiety, and PTSD.

Our findings suggest that SU was related to IVV and STBs, while Mexican immigrants also experienced PTSD symptoms. We also observed a strong association between depression and STBs. Association patterns were replicated by sex and mental health treatment-seeking status. Nevertheless, SU appeared to contribute to Mexican men’s associative model, whereas PTSD symptoms made a major contribution to the Mexican mental health treatment-seeking relationship-pattern model.

These findings underline the need for ongoing efforts through the VDS-UMS and professional primary healthcare system to serve Mexican immigrants in the United States suffering from IVV, SU, mental health symptoms, and STBs. In keeping with Morales-Chainé et al. (6), these findings support continued implementation and standardization of evidence-based screening and brief interventions for interpersonal violence, substance use, and mental health conditions within the Ventanillas de Salud and Mobile Health Units. Expanding these services through the Mexican consular network may help reduce disparities in mental health care access and prevent self-harm and suicidal behaviors among Mexican immigrants in the United States.

### 4.2 Limitations

Although this study provides relevant data on IVV, SU, common mental health presentations, and STBs among Mexican immigrants in the United States, the findings should be interpreted with caution given the cross-sectional design and convenience sampling. Participants were Mexican immigrants who responded to an invitation to receive health psychoeducation through a mental health alliance in the United States; therefore, the sample may represent Mexicans with greater motivation to seek mental health information or care rather than the broader Mexican immigrant population. The sample also included a broad age range, more women than men, and participants residing in several United States, further limiting the generalizability of the findings.

An additional limitation is that we did not assess the level of acculturation or length of residence of the Mexican participants in the United States. These are potentially important unmeasured confounding variables because exposure to United States social and cultural environments over time may be associated with patterns of help-seeking, substance use, exposure to violence, and the expression of common mental health presentations and STBs. Consequently, some of the associations observed among IVV, SU, mental health symptoms, and STBs may vary according to migration and acculturation experiences of Mexican immigrants. Because these factors were not measured, we could not determine whether the structural associations identified in the models were independent of acculturation or duration of residence in the United States.

Furthermore, the findings were based on self-reported measures of violence, SU, mental health symptoms, and STBs, which may have introduced reporting and measurement bias. Future studies should incorporate structured or controlled clinical interviews and recruit more representative samples of Mexican immigrants, including those who are not seeking mental health services. Longitudinal designs should also assess and control for migration-related and social determinants, including age at migration, length of residence in the United States, acculturation or bicultural orientation, language proficiency, documentation status when ethically and safely feasible, sources and timing of violence exposure, and access to social and health services. Including these variables would allow more rigorous examination of whether the observed structural associations remain after accounting for heterogeneity in migration experiences.

## Data Availability

The raw data supporting the conclusions of this article will be made available by the authors, without undue reservation.

## References

[1] Aguilar-Gaxiola S Medina-Mora ME Loera G. "Mental illness and substance use and migration: traumatic exposures during high-risk migration". In: Galea S Ettman C Zaman M, editors. Migration and Health. Chicago, IL: University of Chicago Press (2022). p. 98–107.

[2] Rai A Grossman SF Perkins NH Saasa SK Guzzardo O Tamayo J. Correlates of domestic violence victimization among immigrants during COVID-19 in the United States. J Aggress Maltreat Trauma. (2024) 33:821–43. doi: 10.1080/10926771.2024.2345185

[3] Donlan W Lee J. Coraje, nervios, and susto: culture-bound syndromes and mental health among Mexican immigrants in the United States. Adv Ment Health. (2010) 9:288–302. doi: 10.5172/jamh.9.3.288

[4] Kim W Kim J Cho H Gillis K. Suicide involving intimate partner problems among immigrants in the United States. J Interpers Violence. (2025) 41:1864–85. doi: 10.1177/08862605251322819, 40017458

[5] Taiebine M. Prioritizing mental and brain health in the global response to gender-based violence against women migrants and refugees. Front Public Health. (2025a) 13:1567898. doi: 10.3389/fpubh.2025.1567898, 40124415 PMC11925859

[6] Morales-Chainé S Lira-Chávez IA López-Montoya A Rangel-Gómez MG. "Reducing the attention gap on mental health". In: Gandini L, editor. Migrations. Mexico City: UNAM (2024). p. 262–71.

[7] Sweileh WM. Analysis and mapping of scientific literature on detention and deportation of international immigrants (1990–2022). J Immigr Minor Health. (2023) 25:1065–76. doi: 10.1007/s10903-023-01500-6, 37227605 PMC10209579

[8] Phillimore J Block K Bradby H Darkal H Goodson L Papoutsi A . “I will experience this trauma over and over again”: sexual and gender-based violence, forced migration and structural violence. J Interpers Violence. (2025) 41:3422–52. doi: 10.1177/08862605251338785, 40462264 PMC13139677

[9] Gonzalez FR Benuto LT Casas JB. Prevalence of interpersonal violence among Latinas: a systematic review. Trauma Violence Abuse. (2020) 21:977–90. doi: 10.1177/1524838018806507, 30554556

[10] Cuesta-García A Crespo M. Barriers for help-seeking in female immigrant survivors of intimate partner violence: a systematic review. J Victimol. (2022) 14:33–9. doi: 10.12827/RVJV.14.01

[11] Hindmarch G Meredith LS McCullough CM Griffin BA Watkins KE. Interpersonal violence and mental health, drug use, and treatment utilization among patients with co-occurring opioid use and mental health disorders. Chron. Str. (2024) 8:1–10. doi: 10.1177/24705470241279335, 39246736 PMC11378169

[12] Reyes ME Simpson L Sullivan TP Contractor AA Weiss NH. Intimate partner violence and mental health outcomes among Hispanic women in the United States: a scoping review. Trauma Violence Abuse. (2023) 24:809–27. doi: 10.1177/15248380211043815, 34779327 PMC13110112

[13] Borges G Rafful C Tancredi DJ Saito N Aguilar-Gaxiola S Medina-Mora ME . Mexican immigration to the United States, the occurrence of violence and the impact of mental disorders. Brazil. J. Psychiat. (2013) 35:161–8. doi: 10.1590/1516-4446-2012-0988, 23904022 PMC4164962

[14] Alexander EF Johnson MD. On categorizing intimate partner violence: a systematic review of exploratory clustering and classification studies. J Fam Psychol. (2023) 37:743–52. doi: 10.1037/fam0001075, 36892924

[15] World Health Organization (2026) World Health Organization violence prevention unit: approach, objectives, and activities, 2022–2026. Available online at: https://cdn.who.int/media/docs/default-source/documents/social-determinants-of-health/who_2022_plv_strategy_2022-2026_finalfile.pdf?sfvrsn=c819ff54_3&download=true (Accessed August 17, 2026).

[16] Sabina C Cuevas CA Lannen E. The likelihood of Latino women to seek help in response to interpersonal victimization: an examination of individual, interpersonal and sociocultural influences. Psychosoc Interv. (2014) 23:95–103. doi: 10.1016/j.psi.2014.07.005

[17] Saleem R Pagan-Ortíz ME Morrill Z Brodt M Andrade L. “I thought it would be different”: experiences of structural violence in the lives of undocumented Latinas. Peace Conflict. (2020) 26:171–80. doi: 10.1037/pac0000420

[18] Watcher K Kook-Heffron L Dalpe J. “We weren’t ready”: provider perspectives on addressing intimate partner violence among refugees and immigrants in the United States. J Fam Violence. (2022) 37:235–46. doi: 10.1007/s10896-021-00285-2

[19] Morrison AM Campbell JK Sharpless L Martin SL. Intimate partner violence and immigration in the United States: a systematic review. Trauma Violence Abuse. (2024) 25:846–61. doi: 10.1177/15248380231165690, 37078533 PMC10666473

[20] Eysenbach G. Improving the quality of web surveys: the checklist for reporting results of internet W-surveys (CHERRIES). J Med Internet Res. (2004) 6:e34. doi: 10.2196/jmir.6.3.e34, 15471760 PMC1550605

[21] Weathers F. W. Blake D. D. Schnurr P. P. Kaloupek D. G. Marx B. P. Keane T. M. (2013) The life events checklist for DSM-5 (LEC.5)-standard [measurement instrument]. Life events checklist for DSM-5 (LEC-5)—extended version-fillable form. Available online at: https://www.ptsd.va.gov/professional/assessment/documents/LEC5_Extended_Self-report.PDF (Accessed December 1, 2024).

[22] Weathers F. W. Litz B.T. Keane T. M. Palmieri P. A. Marx B. P. Schnurr P. P. (2013) The PTSD checklist for DSM-5 (PCL-5): LEC-5 and extended criterion A [measurement instrument]. PTSD checklist for DSM-5 (PCL-5)—PTSD: National Center for PTSD. Available online at: https://www.ptsd.va.gov/professional/assessment/adult-sr/ptsd-checklist.asp (Accessed December 1, 2024).

[23] Morales-Chainé S Bacigalupe G Robles-García R López-Fuentes AL Félix-Romero V. Perpetrating–suffering intimate violence: self-harm–suicide thoughts and behaviors, mental health, and alcohol use among Mexican youth during COVID-19. Int J Environ Res Public Health. (2025) 22:955. doi: 10.3390/ijerph22060955, 40566381 PMC12192609

[24] Blevins CA Weathers FW Davis MT Witte TK Domino JL. The posttraumatic stress disorder checklist for DSM-5 (PCL-5): development and initial psychometric evaluation. J Trauma Stress. (2015) 28:489–98. doi: 10.1002/jts.22059, 26606250

[25] Weathers F. W. Litz B. T. Keane T. M. Palmieri P. A. Marx B. P. Schnurr P. P. (2013) The PTSD checklist for DSM-5 (PCL-5)-standard. PTSD checklist for DSM-5 (PCL-5)—fillable form. Available online at: https://www.ptsd.va.gov/professional/assessment/documents/PCL5_Standard_form.PDF (Accessed December 1, 2024).

[26] Goldberg DP Reed GM Robles R Minhas F Razzaque B Fortés S . Screening for anxiety, depression, and anxious depression in primary care: a field study for ICD-11 PHC. J Affect Disord. (2017) 213:199–206. doi: 10.1016/j.jad.2017.02.025, 28278448

[27] Morales-Chainé S Robles-García R López-Montoya A Bosch-Maldonado A Beristain-Aguirre AG Treviño-Santa-Cruz CL . Screening tool for mental health problems during COVID-19 pandemic: psychometrics and associations with sex, grieving, contagion, and seeking psychological care. Front Psychol. (2022) 13:882573. doi: 10.3389/fpsyg.2022.882573 PMC9226722 35756203

[28] World Health Organization. (2010). The alcohol, smoking and substance involvement screening test: manual for use in primary care. Reviewed: the alcohol, smoking and substance involvement screening test (ASSIST). Available online at: https://www.who.int/publications/i/item/978924159938-2 (Accessed December 1, 2024).

[29] Li C. Statistical estimation of structural equation models with a mixture of continuous and categorical observed variables. Behav Res Methods. (2021) 53:2191–213. doi: 10.3758/s13428-021-01547-z 33791955

[30] West SG Wu W McNeish D Savord A. "Model fit in structural equation modeling". In: Hoyle RH, editor. Handbook of Structural Equation Modeling, 2nd Edn. New York, NY: Guilford Press (2023). p. 184–205.

[31] Browne MW Cudeck R. "Alternative ways of assessing model fit". In: Bollen KA Long JS, editors. Testing Structural Equation Models. Newbury Park, CA: Sage Publications (1993). p. 136–62.

[32] Cheug GW Rensvold RB. Evaluating goodness-of-fit indexes for testing measurement invariance. Struct Equ Model. (2002) 9:233–55. doi: 10.1207/S15328007SEM0902_5

[33] R Core Team. R: A Language and Environment for Statistical Computing. Vienna: R Foundation for Statistical Computing (2018).

[34] IBM Corp. IBM SPSS Statistics for Windows, Version 25.0. New York, NY: IBM Corp (2017).

[35] Berenzon-Gorn S Saavedra N Bojorquez I Reed G Wainberg ML Medina-Mora ME. Anxiety among central American migrants in Mexico: a cumulative vulnerability. Int J Environ Res Public Health. (2023) 20:4899. doi: 10.3390/ijerph20064899 PMC10049226 36981807

[36] Taiebine M. A g-local call for culturally responsive prevention and care of gender-based violence against women migrants and refugees. Front Sociol. (2025b) 10:1651018. doi: 10.3389/fsoc.2025.1651018, 41098522 PMC12518089

[37] WHO. mhGAP Intervention Guide for Mental, Neurological and Substance Use Disorders in Non-Specialized Health Settings: Mental Health Gap Action Programme (mhGAP)—Version 2.0. Geneva: WHO (2016). 27786430

