# Supplementary material for: Interpersonal violence and substance use: mental health and self-harm/suicide among Mexicans in VDS-UMS in the United States
Source: Front Public Health. 2026 Oct 8;14:1975732. doi: 10.3389/fpubh.2026.1975732 (PMC13686905; doi:10.3389/fpubh.2026.1975732)
Supplement: Supplementary file 1 [file Data_Sheet_1.docx]

Interpersonal Violence and Substance Use: Mental Health and Self-harm/Suicide among Mexicans in VDS-UMS in the United States

Supplementary File

Appendix A

*Factor Loadings, Standard Errors (SE), and P-value (P) for the Observed Variables for each SEM Variable.*

| **Variables and Measuring Items** | **Loadings** | **SE** | ***p* <** |
| --- | --- | --- | --- |
| **Interpersonal Violence Victimization** | | | |
| Have you been a victim of physical violence (been attacked, hit, slapped, kicked, beaten up)? | 0.567 |  |  |
| Have you been a victim of emotional violence (insults, humiliation, screaming, dismissing, being ignored, or similar experiences)? | 0.681 | 0.115 | 0.001 |
| Have you been a victim of sexual harassment (such as being asked to engage in intimate/physical contact in exchange for academic or work achievements, or experienced continuous verbal-or-online threats)? | 0.705 | 0.140 | 0.001 |
| **STB** | | | |
| Suicidal thought | 0.973 |  |  |
| Suicide plans | 0.951 | 0.028 | 0.001 |
| Suicidal actions | 0.876 | 0.052 | 0.001 |
| **ShTB** | | | |
| Self-harm thoughts | 0.861 |  |  |
| Self-harm plans | 0.913 | 0.026 | 0.001 |
| Self-harm actions | 0.865 | 0.057 | 0.001 |
| **Depression** | | | |
| I want to hurt myself. | 0.590 |  |  |
| I feel little interest or pleasure in activities. | 0.798 | 0.179 | 0.001 |
| I have felt down, depressed, or hopeless. | 0.862 | 0.208 | 0.001 |
| **Anxiety** | | | |
| I have felt nervous or on edge | 0.839 |  |  |
| I have felt unable to control my worrying | 0.865 | 0.070 | 0.001 |
| I have felt so restless it was hard to keep still | 0.798 | 0.068 | 0.001 |
| I have had trouble relaxing | 0.861 | 0.068 | 0.001 |
| I have felt afraid something awful could happen | 0.812 | 0.085 | 0.001 |
| **Re-experiencing** | | | |
| Repeated, disturbing, and unwanted memories of the stressful experience | 0.922 |  |  |
| Feeling very upset when something reminded you of the stressful experience | 0.979 | 0.05 | 0.001 |
| **Avoidance** | | | |
| Avoiding memories, thoughts, or feelings related to the stressful experience | 0.916 |  |  |
| Avoiding external reminders of the stressful experience (such as people, places, conversations, activities, objects, or situations) | 0.914 | 0.056 | 0.001 |
| **NACM** | | | |
| Having strong negative beliefs about yourself, other people, or the world (for example, having thoughts such as “I am bad,” “There is something seriously wrong with me,” “No-one can be trusted,” “The world is completely dangerous”) | 0.875 |  |  |
| Loss of interest in activities you used to enjoy | 0.891 | 0.068 | 0.001 |
| **Hyperarousal** | | | |
| Feeling jumpy or easily startled | 0.902 |  |  |
| Having difficulty concentrating | 0.923 | 0.062 | 0.001 |
| **SU**  **In your life, which of the following substances have you *ever used* (non-medical use only)?** | | | |
| Tobacco products (cigarettes, chewing tobacco, cigars, etc.) | 0.799 |  |  |
| Alcoholic beverages (beer, wine, spirits) | 0.700 | 0.082 | 0.001 |
| Cannabis (marijuana, pot, grass, or hash) | 0.887 | 0.065 | 0.001 |
| Cocaine (coke, crack etc.) | 0.919 | 0.065 | 0.001 |
| Amphetamine-type stimulants (speed, meth, ecstasy, etc.) | 0.868 | 0.071 | 0.001 |
| Inhalants (nitrous, glue, petrol, paint thinner, etc.) | 0.846 | 0.093 | 0.001 |
| Sedatives or sleeping pills (such as diazepam, alprazolam, flunitrazepam, or midazolam, etc.) | 0.696 | 0.079 | 0.001 |
| Hallucinogens (LSD, acid, mushrooms, trips, ketamine, etc.) | 0.808 | 0.077 | 0.001 |
| Opioids (heroin, morphine, methadone, buprenorphine, codeine, etc.) | 0.855 | 0.129 | 0.001 |
| Other | 0.776 | 0.116 | 0.001 |

*Note*. STB = Suicidal Thoughts and behaviors. ShTB = Self-harm Thoughts and Behaviors. NACM = Negative alterations in cognitions and mood, SU = Lifetime Substance Use.

Appendix B

*Chi-square Analysis, Degrees of Freedom, P-values, Fit Indices, and Cronbach’s Alpha by Scales for all Participants*

| **Scales** | **X²** | **df** | ***p*≤** | **RMSEA** | **Confidence Interval** | **SRMR** | **CFI** | **TLI** | **Cronbach´s alpha** |
| --- | --- | --- | --- | --- | --- | --- | --- | --- | --- |
| LEC-5 Interpersonal Violence Victimization | 0.000 | 0 | 0.000 | 0.000 | 0.000-0.000 |  | 1.000 | 1.000 | 0.53 |
| mhGAP STBs | 27.752 | 8 | 0.001 | 0.053 | 0.032-0.074 |  | 0.997 | 0.994 | 0.85 |
| Depression and Anxiety | 69.228 | 18 | 0.000 | 0.056 | 0.043-0.071 | 0.015 | 0.990 | 0.985 | 0.93 |
| PCL-5 Brief Version | 57.776 | 14 | 0.000 | 0.059 | 0.044-0.075 | 0.009 | 0.995 | 0.990 | 0.97 |
| ASSIST SU | 95.662 | 35 | 0.000 | 0.044 | 0.034-0.055 |  | 0.981 | 0.975 | 0.75 |
| *Note*. LEC-5 = Life Events Checklist, mhGAP-mental health Gap Action Programme, STBs = Self-harm and Suicidal Thoughts and Behaviors, PCL-5 = Posttraumatic Checklist 5th Version, ASSIST = Alcohol, Smoking, and Substance Involvement Screening Test. SU = Lifetime Substance Use. Categorical Variables do not show SRMR as recommended by Li (2021). | | | | | | | | | |

Appendix C

*Residuals of the Covariance Matrix from the Model of the Whole Sample*

|  | Interpersonal Violence Victimization | STB | ShTB | Depression | Anxiety | Reexperiencing | Avoidance | NACM | Hyperarousal | SU |
| --- | --- | --- | --- | --- | --- | --- | --- | --- | --- | --- |
| Interpersonal Violence Victimization | 0.322 |  |  |  |  |  |  |  |  |  |
| STB | 0.369 | 0.947 |  |  |  |  |  |  |  |  |
| ShTB | 0.326 | 0.912 | 0.740 |  |  |  |  |  |  |  |
| Depression | 0.614 | 1.059 | 0.942 | 1.889 |  |  |  |  |  |  |
| Anxiety | 1.427 | 1.914 | 1.695 | 3.282 | 9.069 |  |  |  |  |  |
| Reexperiencing | 0.492 | 0.435 | 0.405 | 0.939 | 2.181 | 1.789 |  |  |  |  |
| Avoidance | 0.469 | 0.364 | 0.341 | 0.895 | 2.079 | 1.629 | 1.585 |  |  |  |
| NACM | 0.566 | 0.498 | 0.422 | 1.080 | 2.510 | 1.471 | 1.439 | 1.565 |  |  |
| Hyperarousal | 0.553 | 0.490 | 0.472 | 1.055 | 2.451 | 1.557 | 1.482 | 1.510 | 1.586 |  |
| SU | 0.123 | 0.460 | 0.376 | 0.234 | 0.544 | 0.187 | 0.179 | 0.216 | 0.210 | 0.639 |

*Note*. STB = Suicide Thoughts and Behaviors, ShTB = Self-harm Thoughts and Behaviors, NACM = Negative Alterations in Cognitions and Mood, SU = Lifetime Substance Use.
